# Usefulness of rapid on-site evaluation specimens from endoscopic ultrasound-guided fine-needle aspiration for cancer gene panel testing: A retrospective study

**DOI:** 10.1371/journal.pone.0228565

**Published:** 2020-01-30

**Authors:** Tetsuya Ishizawa, Naohiko Makino, Akiko Matsuda, Yasuharu Kakizaki, Toshikazu Kobayashi, Chisaki Ikeda, Shinpei Sugahara, Michihiko Tsunoda, Yoshiyuki Ueno

**Affiliations:** Department of Gastroenterology, Faculty of Medicine, Yamagata University, Yamagata, Japan; Universita degli Studi di Napoli Federico II, ITALY

## Abstract

Pancreatic cancer (PC) is a highly lethal malignancy, with a 5-year survival rate of 6%. Cancer gene panel testing is expected to allow selection of suitable therapeutic drugs in individual patients with PC and improve their prognosis. Although somatic mutations can be identified in formalin-fixed, paraffin-embedded samples derived from surgical specimen, the rate of surgical indication among patients with PC is only 20%. To acquire genome information with a less invasive method, we used rapid on-site evaluation (ROSE) specimens from endoscopic ultrasound-guided fine-needle aspiration. The present study aimed to retrospectively evaluate the utility of comprehensive cancer gene panel testing with ROSE specimens. DNA was extracted from preserved ROSE specimens of 26 patients diagnosed with PC between 2011 and 2017. DNA sequences of oncogenes and cancer-related genes were determined using the Ion AmpliSeq Comprehensive Caner Panel. We compared *KRAS* mutations between cancer gene panel testing by next-generation sequencing (NGS) and *KRAS* mutation analysis by polymerase chain reaction. The mean yield of DNA per extraction from ROSE specimens was 171 ng (range, 34–478 ng). On cancer gene panel testing, we noted *KRAS* mutations (92%), *TP53* mutations (50%), *CDKN2A* mutations (15%), and *SMAD4* mutations (31%). The concordance rate of *KRAS* mutations between cancer gene panel testing by NGS using ROSE specimens and *KRAS* mutation analysis by the companion diagnostics using residual materials was 81%. Among five cases of *KRAS* discordance, three showed *KRAS* mutations in cancer gene panel testing but not in *KRAS* mutation analysis. Cancer gene panel testing with ROSE specimens can help stratify unresectable PC patients without additional invasive approaches, and it can be used for therapeutic drug selection.

## Introduction

Pancreatic cancer (PC) is a highly lethal malignancy, with a 5-year survival rate of 6% [1], and it has been predicted to be the second leading cause of cancer mortality in the USA by 2030 [2]. Only 20% of patients are considered suitable for surgical resection, which is the only curative therapy for PC, and the remaining 80% of patients are treated with non-surgical approaches, such as chemotherapy [3]. The first choice of chemotherapy has been FOLFIRINOX (oxaliplatin, irinotecan, fluorouracil, and leucovorin) or gemcitabine plus nab-paclitaxel; however, the median overall survival with FOLFIRINOX was reported to be 11.1 months [4] and that with gemcitabine plus nab-paclitaxel was reported to be 8.5 months [5].

In recent years, the development of precision medicine has changed the treatment of many cancers, such as breast cancer, melanoma, colorectal cancer, and lung cancer [6]. Although *KRAS*, *TP53*, *CDKN2A*, and *SMAD4* mutations are representative gene mutations of PC [7] [8], in many cases, 12 core signaling pathways have been shown to be potential therapeutic targets for precision medicine [9]. Several studies have reported successful clinical cases where stratification therapy was implemented for PC [10–14]. Solid tumors with mismatch-repair deficiency, including PC, have been reported to be sensitive to immune checkpoint blockade with anti-PD-1 antibodies [10]. The administration of maintenance olaparib, which is a poly(adenosine diphosphate-ribose) inhibitor, for patients with germline *BRCA*-mutated metastatic PC has been reported to prolong progression-free survival [11].

Although germline mutations can be assessed using peripheral blood, in cases of somatic mutations, which are therapeutic targets of many drugs, mutation assessment in formalin-fixed, paraffin-embedded (FFPE) samples derived from surgical specimens is the gold standard. However, the rate of surgical indication among patients with PC has been reported to be only 20% [3], and the approach for assessing target mutations in the remaining 80% of patients is unclear. Some studies have reported that genetic tests using cytological specimens and liquid biopsy are useful [15, 16].

To acquire genome information with a less invasive method, we used rapid on-site evaluation (ROSE) specimens from endoscopic ultrasound-guided fine-needle aspiration (EUS-FNA). The present study aimed to retrospectively evaluate the utility of comprehensive cancer gene panel testing with ROSE specimens.

## Materials and methods

### Patients

We reviewed our hospital’s EUS-FNA database between January 2011 and December 2017, and searched for patients who met the following criteria: (1) suspected with PC, (2) had suspicious or positive findings on cytology, (3) underwent ROSE, and (4) underwent *KRAS* mutation analysis. We identified 26 patients and confirmed from electronic medical records that each patient was diagnosed histologically or clinically with PC. We represented the age of the patient group as mean, minimum, and maximum and other clinical information as percentages.

### Endoscopic ultrasound-guided fine-needle aspiration

EUS-FNA was performed with a 22-gauge needle (EZ shot 2, Olympus, Corp., Tokyo, Japan; EchoTip Procore, COOK medical, Inc, Bloomington, IN, USA) using a linear echoendoscope (UCT240AL5 or UCT260, Olympus, Corp., Tokyo, Japan). After the aspirated materials were placed onto a petri dish using a stylet, the white tissues that were likely to contain a lot of pancreatic tissue were placed in formalin solution for pathological analysis, and the red tissues that were likely to contain a lot of blood were divided on two glass slides using a fitting method. One slide was stained with rapid hematoxylin and eosin for ROSE, and the other was fixed with alcohol for subsequent Papanicolaou staining. The needle catheter was flushed with 2 mL of saline, and residual materials were collected and placed into 2-mL tubes for *KRAS* mutation analysis [17]. In ROSE, the quality of the aspirated materials was assessed by a cytologist in a room. Residual materials were frozen and sent to a clinical testing company, and after DNA extraction, *KRAS* mutation analysis was performed using the Scorpion amplified refractory mutation system (scorpion-ARMS) method (BML, Inc., Tokyo, Japan) [18] or the polymerase chain reaction–reverse sequence specific oligonucleotide (PCR-rSSO) method (SRL, Inc., Tokyo, Japan) [19].

### DNA extraction

ROSE slides were immersed in xylene for 2 days until the coverslip detached. Following rinsing in 95% ethanol, all cellular materials were scraped with a sterile razor and placed into 1.5-mL tubes [15]. DNA was extracted using a DNA extraction kit (DNA micro-kit, Qiagen, Hilden, Germany) and a fully automatic nucleic acid purification system (QIA cube, Qiagen), according to the manufacturer’s instructions. The elution volume was set to 20 μL. In 1 μL of DNA extract, the DNA concentration (ng/μL) was measured using the digital TapeStation System with Genomic Screen Tape (2200 TapeStation, Agilent Technologies, Palo Alto, CA, USA). We represented DNA yield as mean, minimum, and maximum. The plot of DNA yield was constructed using the R-software beeswarm package version 0.2.3. ROSE-derived samples containing 20 ng of gDNA were used for amplicon-based NGS.

### Next-generation sequencing

In cancer gene panel sequencing, libraries were constructed using an amplicon-based system (Ion AmpliSeq Library Kit v2.0, Thermo Fisher Scientific, Waltham, MA, USA), according to the manufacturer’s instructions. Quantification of the libraries was performed using the digital TapeStation system with D1000 Screen Tape (2200 TapeStation, Agilent Technologies). After assessing the quality of the libraries, amplified libraries were subjected to emulsion PCR using a commercial Ion AmpliSeq Comprehensive Cancer Panel^™^ (Thermo Fisher Scientific, Waltham, MA, USA), which targets the all-exon coverage of 409 cancer and cancer-related genes (S1 Table), and sequencing was performed with PI chip v3 using the Ion Proton next-generation sequencer (Thermo Fisher Scientific). Genome assembly from FASTQ sequencing files and variant calling from BAM files were performed using our bioinformatics pipeline through mapping to human genome reference sequence (hg38, https://genome.ucsc.edu/index.html) with BWA (http://bio-bwa.sourceforge.net/bwa.shtml) and Bowtie2 (http://bowtie-bio.sourceforge.net/bowtie2/index.shtml) programs, variant calling with the Freebayes program (https://github.com/ekg/freebayes), and variant annotation with the ANNOVAR program (http://annovar.openbioinformatics.org). Filtering variants performed with these conditions, the depth of sequencing coverage >20, base/mapping quality ≥10, allele fraction ≥0.02, and the use of duplicate reads. Nonsynonymous, nonsense mutations were manually reviewed and interpreted. We represented the read depth, effective read rate, and discordance rate as mean, minimum, and maximum and other results as percentages.

### Ethics

This retrospective study was approved by the Institutional Review Board of Yamagata University Faculty of Medicine (IRB H29-294). We completely anonymized all samples before accessing them. We accessed all samples between October 2017 and January 2018. All samples were sourced from Yamagata University Hospital.

## Results

### Clinicopathological characteristics

Comprehensive cancer gene panel testing by NGS was performed using ROSE specimens in 26 patients. The mean patient age was 66 years (range, 43–81 years), and the male-to-female ratio was 1:1. Among the study patients, 6 (23%) had a family history of PC and 1 (4%) had a history of intraductal papillary mucinous neoplasm. Additionally, 17 patients (65%) were Ca19-9 positive, and 22 patients (85%) had clinical stage III or IV disease. Moreover, 17 patients (65%) had positive findings on cytological analysis of EUS-FNA samples, and 9 patients (35%) had suspicious findings. Furthermore, 23 patients (89%) had *KRAS* mutations on KRAS mutation analysis using residual materials from EUS-FNA.

### Cancer gene panel testing

**Fig 1:**
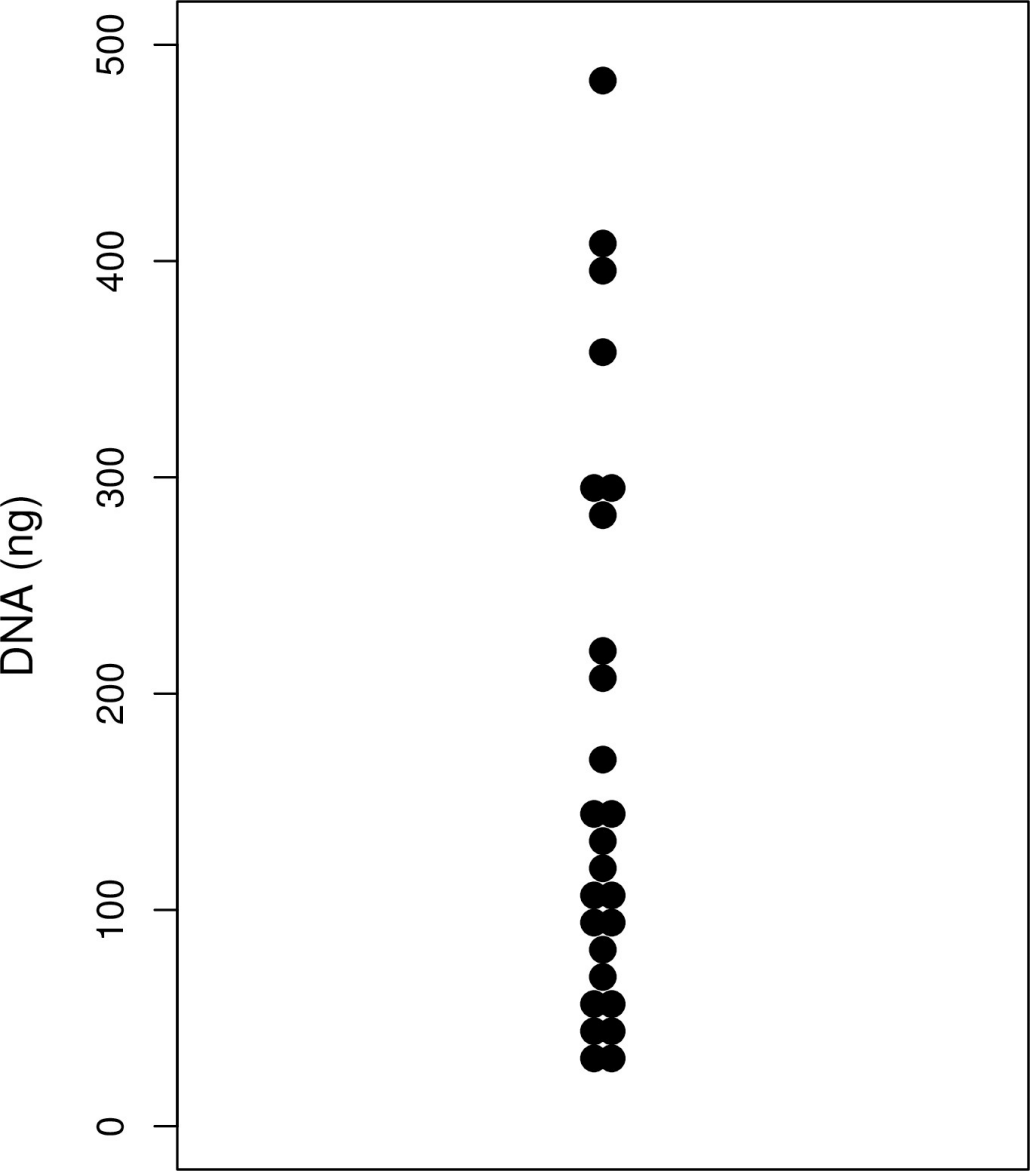
The amount of ROSE-derived sample DNA.

**Fig 2:**
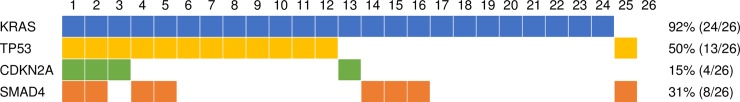
The detection rates of mutations of *KRAS*, *TP53*, *CDKN2A*, and *SMAD4*.

The mean yield of DNA per extraction was 171 ng (range, 34–478 ng) (Fig 1). The mean read depth of cancer gene panel testing by NGS was 828 (range, 396–2222), mean effective read rate was 0.97 (range, 0.93–0.98), and mean discordance rate was 0.0018 (range, 0.0012–0.0028). The rate of *KRAS* mutations was 92% (24/26), *TP53* mutations was 50% (13/26), *CDKN2A* mutations was 15% (4/26), and *SMAD4* mutations was 31% (8/26) on cancer gene panel testing (Fig 2 and Table 1).

**Table 1:** The details of the detected mutations.

Gene	Variant (n)	COSMIC^a^ ID (count)	ExAC[20]^b^	GERP[21]^c^	CLNSIG^d^
**KRAS**	G12C (1)	1140136 (5069)	0.00001142	5.68	Pathogenic
	G12D (7)	1135366 (14861)	0.00002283	5.68	Pathogenic
	G12R (5)	1157797 (1427)	NA	5.68	Pathogenic
	G12V (10)	520 (10163)	NA	5.68	Pathogenic
	Q61H (1)	555 (109)	NA	5.77	Pathogenic
**TP53**	V41M (1)	11084 (88)	NA	5.59	Pathogenic
	R43H (2)	10648 (1509)	9.42E-06	5.41	Pathogenic
	A57V (1)	5095505 (6)	4.71E-05	5.41	Uncertain Significance
	L91F (2)	1649369 (21)	NA	5.48	Uncertain Significance
	Y102N (1)	4271828 (25)	NA	4.62	Likely Pathogenic
	C106Y (1)	1649400 (131)	9.42E-06	4.09	Pathogenic
	C110F (1)	129834 (112)	NA	4.62	Uncertain Significance
	V140M (1)	3388172 (131)	3.08E-05	5.13	Uncertain Significance
	R141H (1)	99729 (996)	3.02E-05	4.92	Pathogenic
	C143Y (1)	165084 (81)	NA	4.92	Pathogenic
	E153K (1)	137087 (209)	NA	4.99	Pathogenic
**CDKN2A**	R58X (1)	99730 (167)	NA	2.71	Pathogenic
	P81L (1)	3092302 (22)	NA	5.93	NA
	W110X (1)	3382498 (34)	NA	5.01	Pathogenic
	L130Q (1)	3395738 (7)	NA	5.77	NA
**SMAD4**	R135X (1)	14168 (27)	NA	4.57	Pathogenic
	D351Y (1)	1151549 (8)	NA	5.86	NA
	P356S (1)	1226726 (7)	NA	5.86	NA
	R361C (1)	14140 (108)	NA	5.86	Pathogenic
	R361H (1)	14122 (128)	NA	5.86	Pathogenic
	G386D (1)	1150895 (21)	NA	5.65	Pathogenic
	A406T (1)	14103 (9)	NA	5.49	Uncertain Significance
	C499Y (2)	14221 (7)	NA	6.08	NA

^a^COSMIC https://cancer.sanger.ac.uk/cosmic

^b^ExAC http://exac.broadinstitute.org/

^c^GERP http://mendel.stanford.edu/SidowLab/downloads/gerp/

^d^CLNSIG http://annovar.openbioinformatics.org/

**Fig 3:**
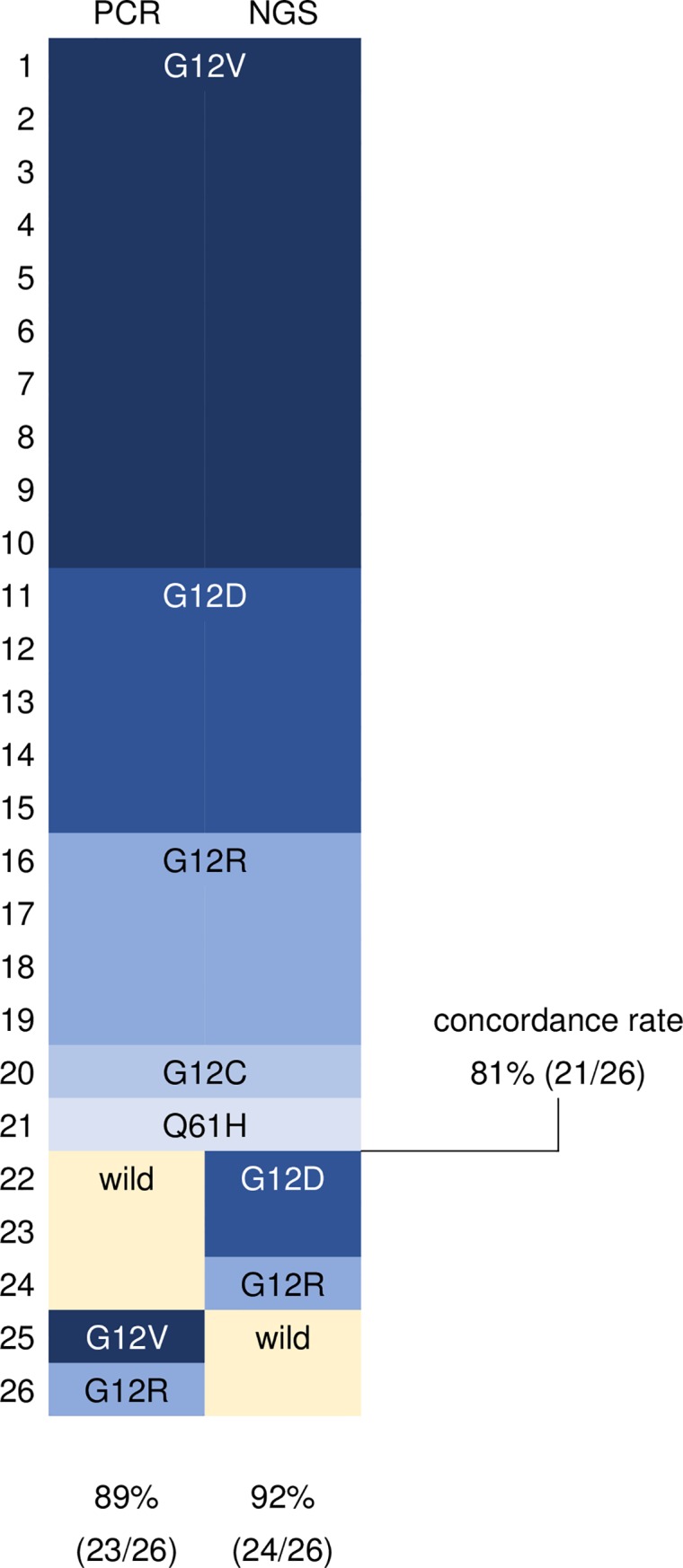
The concordance rate of *KRAS* mutations.

The concordance rate of *KRAS* mutations between cancer gene panel testing by NGS using ROSE specimens and *KRAS* mutation analysis by the Scorpion-ARMS or PCR-rSSO method using residual materials was 81% (21/26) (Fig 3). Among the five cases of *KRAS* discordance, three showed *KRAS* mutations in cancer gene panel testing but not in *KRAS* mutation analysis and two showed *KRAS* mutations in *KRAS* mutation analysis but not in cancer gene panel testing. Two cases of *KRAS* wild-type in cancer gene panel testing showed *KRAS* mutations on viewing the BAM file (KRAS G12V, allele frequency 1%; KRAS G12R, allele frequency 1%), and these findings were concordant with the results of *KRAS* mutation analysis using residual materials.

## Discussion

EUS-FNA could help in the diagnosis of pancreatic lesions, and the use of ROSE could increase the diagnostic yield of EUS-FNA by 10%–30% [22]. ROSE specimens used in this study had three features. First, ROSE specimens at our institution were created by rapid hematoxylin and eosin staining, which has been reported to provide almost the same findings as rapid Papanicolaou staining [23] and has been reported not to affect DNA analysis [24]. Second, ROSE specimens at our institution were evaluated by a cytotechnologist to assess sample adequacy. A previous prospective double-blind study showed that specimen adequacy was more accurately assessed by a cytotechnologist than by an experienced endosonographer [25]. Third, ROSE specimens used in this study were prepared by the fitting method with two glass slides and were confirmed to be suspicious or positive on cytological analysis with Papanicolaou staining. Therefore, we consider that our ROSE specimens are appropriate for use in cancer gene panel testing.

Previous studies comparing cytology and FFPE samples as sources of DNA reported that the mutated *KRAS* detection rate was higher with cytology samples than with FFPE samples (77% vs. 57%) [26] and that NGS could be performed with 5- to 8-fold less input DNA when using cytology samples than when using FFPE samples [27]. Considerable evidence suggests that formaldehyde induces DNA degradation [28]. Furthermore, cytology samples have been reported to have the potential to concentrate tumor cells, because less cohesive tumor cells might be extracted [15]. Therefore, we used ROSE specimens, which are cytology samples.

Several studies have reported the usefulness of cancer gene panel testing with EUS-FNA-derived specimens. Kameta et al. performed cancer gene panel testing of 50 genes using EUS-FNA-derived frozen specimens for pancreatic cancer (n = 27) and detected *KRAS* mutations (96%), *TP53* mutations (44%), *CDKN2A* mutations (11%), and *SMAD4* mutations (7%), and they confirmed *KRAS* mutations using TaqMan PCR analysis [29]. Gleeson et al. performed cancer gene panel testing of 160 genes using EUS-FNA-derived cytology specimens for pancreatic cancer (n = 29) and detected *KRAS* mutations (93%), *TP53* mutations (72%), *CDKN2A* mutations (0%), and *SMAD4* mutations (31%), and the mutations detected from EUS-FNA-derived cytology were consistent with the mutations detected from surgical specimens [15]. However, we believed that the use of frozen specimens would increase the number of punctures, making it difficult to confirm the presence or absence of cancer cells. In addition, we considered that the use of cytology would have the problem of loss of diagnostic material. Recently, the usefulness of digital slides for biopsy specimens has been reported [30, 31]; however, these reports did not include cytology specimens. The problems with digital slides for thick cytology specimens are assessment difficulty, large file size, and high time requirement [32]. Thus, the use of ROSE specimens allowed confirmation of the presence or absence of cancer cells without increasing the number of punctures and allowed cancer gene panel testing, leaving diagnostic material.

Two cases of KRAS wild-type in the cancer panel examination appeared to have insufficient cancer cell content in ROSE. Using a puncture needle, such as the franseen biopsy needle [33], for collecting a larger amount of the sample would increase the cancer cell content and reduce false negatives.

The present study has several limitations. First, comparisons were not performed with surgical specimens. The surgical specimens at our institution were not suitable for DNA analysis because the formalin-fixation period was long. Second, comparisons were not performed with EUS-FNA-derived FFPE specimens. The EUS-FNA-derived FFPE specimens at our institution were not suitable because the number of cancer cells was limited. Third, the sample size was small.

ROSE specimens can be used as not only rapid cytology samples but also cancer genome sources for stratified treatment. Cancer gene panel testing with ROSE specimens can help stratify unresectable PC patients without additional invasive approaches, and it can be used for therapeutic drug selection.

## Supporting information

S1 Table Target gene list of the Ion AmpliSeq Comprehensive Cancer Panel^™^. This panel targets the all-exon coverage of 409 cancer and cancer-related genes.
(XLSX)
